# Emotional crisis in a naturalistic context: characterizing outpatient profiles and treatment effectiveness

**DOI:** 10.1186/s12888-017-1293-3

**Published:** 2017-04-07

**Authors:** Adriano Zanello, Laurent Berthoud, Jean-Pierre Bacchetta

**Affiliations:** 1 Department of Mental Health and Psychiatry, University Hospitals of Geneva, Geneva, Switzerland; 2 Department of Psychiatry, University of Lausanne, Lausanne, Switzerland; 3 HUG Département de Santé Mentale et de Psychiatrie, Site Belle-Idée, Ch. du Petit-Bel-Air 2, CH-1225 Chêne-Bourg, Switzerland

**Keywords:** Crisis intervention, Brief psychiatric rating scale, Defense mechanisms, Recovery style, Community mental health services, Treatment effectiveness

## Abstract

**Background:**

Crisis happens daily yet its understanding is often limited, even in the field of psychiatry. Indeed, a challenge is to assess the potential for change of patients so as to offer appropriate therapeutic interventions and enhance treatment program efficacy. This naturalistic study aimed to identify the socio-demographical characteristics and clinical profiles at admission of patients referred to a specialized Crisis Intervention Center (CIC) and to examine the effectiveness of the intervention.

**Method:**

The sample was composed of 352 adult outpatients recruited among the referrals to the CIC. Assessment completed at admission and at discharge examined psychiatric symptoms, defense mechanisms, recovery styles and global functioning. The crisis intervention consisted in a psychodynamically oriented multimodal approach associated with medication.

**Results:**

Regarding the clinical profiles at intake, patients were middle-aged (M = 38.56, SD = 10.91), with a higher proportion of women (62.22%). They were addressed to the CIC because they had attempted to commit suicide or had suicidal ideation or presented depressed mood related to interpersonal difficulties. No statistical differences were found between patients dropping out (*n* = 215) and those attending the crisis intervention (*n* = 137). Crisis intervention demonstrated a beneficial effect (*p* < 0.01) on almost all variables, with Effect Sizes (ES) ranging from small to large (0.12 < ES < 0.75; median = 0.49). However, the Reliable Change Index indicated that most of the issues fall into the undetermined category (range 41.46 to 96.35%; median = 66.20%).

**Conclusions:**

This study establishes the profile of patients referred to the CIC and shows that more than half of the patients dropped out from the crisis intervention before completion. Our findings suggest that people presenting an emotional crisis benefit from crisis intervention. However, given methodological constraints, these results need to be considered with caution. Moreover, the clinical significance of the improvements is not confirmed. Thus, the effectiveness of crisis intervention in naturalistic context is not fully determined and should be more rigorously studied in future research.

**Electronic supplementary material:**

The online version of this article (doi:10.1186/s12888-017-1293-3) contains supplementary material, which is available to authorized users.

## Background

Crisis is a configuration involving a precipitating event, an emotional response and an intervention [1]. The nature of crisis events may be either public, as with natural disasters or terrorist attacks, or private, such as the notification of a chronic illness, domestic violence, loss of the sense of self, or sexual abuse [2]. Public crisis events are obvious, perceived and even shared by others, e.g. SARS crisis [3]. On the other hand, private events are often hidden to others who may be unaware of what is going on [4]. The way people perceive and experience the event may lead to a crisis response. The latter is an individual reaction to stress with the potential to produce psychopathological symptoms [5]. It is also considered as a transition phase that may change behaviors and interpersonal functioning [6]. Crisis interventions are thought to provide acute psychological care enabling people in crisis to recover their adaptive level of functioning and to circumvent potential deleterious effects of the psychological trauma [7].

Among crisis interventions one can find psychodynamic, behavioral, cognitive-behavioral, developmental, systems, radical and social construction approaches as well as eye movement desensitization and reprocessing (EMDR) and strategic solution-focused therapy, crisis incident and stress management for specific crises [4, 7, 8]. These treatments are delivered generally during 2 months by well-trained staff members (e.g. psychiatrists, nurses, psychologists, social workers...) in specific structures such as Crisis Resolution Teams [9, 10], Women’s Crisis Houses [11], Crisis Intervention Units [12] or Crisis Intervention Centers [13–17]. Even though there are important differences among services and clinical practices, crisis interventions share some characteristics - such as administering rapid and time-limited short-term interventions (< 3 months), being a 24/7 service, offering a helpline - that provide an alternative to psychiatric hospitalization, facilitate discharge, play a gatekeeping function regarding inpatient units, involve multidisciplinary teams, and aim to stabilize individuals facing a crisis. Crisis interventions also share common ingredients in treatment success, namely “therapist warmth, and empathy, the therapeutic alliance, insight and feedback and action factors such as cognitive mastery” (p. 18) [2]. One of the challenges in crisis intervention is to seize the potential for change while giving patients enough support and security as well as maintaining social integration [15]. Therefore, it is important to carefully identify the patient’s characteristics at admission in specific crisis structures, in order to offer appropriate therapeutic interventions and improve the efficacy of the treatment programs [18]. On the other hand, it is also crucial, for patients, relatives, caregivers, hospital policy, and social insurances to document crisis interventions efficiency. Several studies have examined the efficiency of the specific treatments administered in various crisis center contexts. Most of the research examined whether Crisis Intervention contributed to reducing the global rate of psychiatric hospitalization and readmission in emergency units to cost-effectiveness, and enhanced client satisfaction [9, 10, 15, 16, 19–22]. A meta-analysis comprised of 36 crisis intervention studies yielded large effect sizes [2]. Unfortunately, the authors did not specify the measures included. Only few studies have reported Crisis Intervention effects on symptoms, social functioning and quality of life, showing a positive short term effect on these variables [23–25], while in the long term findings remain controversial [14].

Several authors have claimed that the central characteristic of crisis is its potential for change in the psychological functioning of patients [6, 13, 26–28]. Moreover, within the framework of psychodynamic theory, it is hypothesized that patients preferentially use maladaptive defense mechanisms during emotional crisis [27]. To the best of our knowledge, no empirical study has examined these aspects of crisis. Thus, it seems that “… research on crisis intervention is in its early stage of development” (p.18) [2].

This led us to design a clinical prospective naturalistic study, which is more suitable to complex interventions, such as those taking place in the CICs, rather than randomized research [29, 30]. The aims of this study were a) to present the functioning of a CIC, b) to characterize the crisis context, socio-demographic, psychiatric and clinical profile of the patients referred to the CIC and c) to assess the effectiveness of a psychodynamically oriented [27] crisis intervention.

## Methods

In Geneva, the CIC was introduced as an alternative to hospitalization [13] as part of psychiatry sectorization. Referrals to the CIC are made by the patients themselves or by concerned others such as private practitioners (e.g. psychiatrists, psychotherapists, physicians…), the emergency unit of the General Hospital, specialized mental units (e.g. affective disorders, eating disorders, personality disorders, substances abuse units…), and outpatient and inpatient units of the Psychiatric Hospital.

The CIC team is multidisciplinary. It offers three main services: clinical assessment, time-limited support therapy, and crisis intervention either in individual or group format. If necessary, patients can be offered one of the eight crisis beds in the center for up to seven nights.

Admission to the CIC is done in the 24 h after the initial call. Patients participating in the crisis program are assigned to a psychiatrist and a nurse; this duo remains stable throughout the intervention. Patients participating in the crisis intervention are proposed individual and group therapy as well as support by social workers, and family or couple interventions [15]. Crisis intervention generally lasts 6–8 weeks [27], but its length can be tailored to the patient’s specific needs**.** The assigned duo decides with the patient when to end the program.

### Patients

Outpatients were recruited among the referrals to one of the four CICs of the Department of Psychiatry, which is part of the Geneva University Hospitals in Switzerland, from 2005 to 2010. Inclusion criteria were: being 18 to 65 years old, fulfilling an ICD-10 diagnosis for mental disorders, presenting an emotional crisis, and participating in the intensive crisis intervention program [15]. Exclusion criteria were: difficulties in spoken or written French, behaviors that could not allow assessment (e.g. hostility, disorganization, under the influence of substance abuse), suspicion of or documented neurocognitive alterations, or mental retardation.

### Measures

Measures at admission included socio-demographic information, crisis details and clinical variables. Patients participated in a semi-structured interview assessing psychiatric symptom severity and completed a set of self-administered questionnaires measuring psychiatric symptoms, defense mechanisms, recovery styles and global functioning.

#### Symptoms

Psychiatric symptoms were assessed with two tools, the Brief Psychiatric Rating Scale expanded version 4.0 (BPRS 4.0) [31] (Zanello et al., Adaptation française de la Brief Psychiatric Rating Scale version 4.0, unpublished) and the Symptom Checklist 90 - Revised (SCL-90-R) [32, 33]. The BPRS 4.0 is a semi-directive interview composed of 24 items rated from 1 (absent) to 7 (severe). In order to have a general representation of symptom severity, and because no calibration of the BPRS 4.0 to equivalent clinical judgments exists, we borrowed and modified the categories defined by Brenner’s group [34]. We considered at intake five categories of general distress defined as follows: no illness (all items scores <3), mild severity (at least one item =3, but no item ≥4), moderate (at least one item =4, but no item ≥5), severe (at least one item =5, but no item ≥6), and extremely severe (at least one item ≥6). This procedure was used because low BPRS 4.0 scores can be obtained even if only one item is rated extremely severe (score = 7) while the others are absent (score = 1). This variable was labeled BPRS-4.0 Categories (BPRS 4.0-C). Changes occurring after CIC intervention were examined comparing the BPRS 4.0 mean score of reality distortion, apathy, activity, mood disturbance, disorganization and somatization dimensions [35], obtained at intake and at discharge.

The SCL-90-R is a self-rating scale composed of 90 items rated from 0 (not at all) to 4 (extremely). At intake, we considered the Global Severity Index (GSI) and transformed it into T-scores (mean = 50, SD = 10) found in published norms [36]. Borrowing the guidelines for interpreting T scores [37], we considered five categories defined as follows: no distress (*T* ≤ 55), mild (56 ≤ *T* < 60), moderate (61 ≤ *T* < 65), severe (65 ≤ *T* < 70), and extremely severe (*T* ≥ 70). This variable was labelled SCL-90-R Categories (SCL-90-R-C). Possible changes due to crisis intervention were examined comparing the scores at intake and at discharge on the nine symptoms dimensions of SCL-90-R: Somatisation, Obsessive-compulsivity, Interpersonal sensitivity, Depression, Anxiety, Hostility, Phobic anxiety, Paranoid ideation and Psychoticism.

#### Defense mechanisms and recovery styles

As emotional crisis leads to a breakdown in defense mechanisms [27], the latter were examined with the Defense Style Questionnaire (DSQ 40) [38, 39]. The DSQ 40 is a self-reported questionnaire composed of 40 items assessed on a 9-point Likert scale. It measures 20 defense mechanisms, each being represented by 2 items. These mechanisms are grouped as being adaptive (mature defense: e.g. humor, suppression, sublimation), intermediate (neurotic defense: e.g. undoing, idealization, reaction formation), or maladaptive (immature defense: e.g. splitting, projection, acting out). A mean score was calculated for each of these 3 dimensions.

The Recovery Style Questionnaire (RSQ) [40] (Zanello and Koellner, Adaptation française du Recovery Style Questionnaire, unpublished) assesses the patients’ attitude about their mental illness. This attitude is underpinned by the mechanisms of defense. The RSQ is a self-reported questionnaire composed of 39 yes-no items. It assesses on a continuum two opposite main recovery styles identified as integration and sealing-over [41]. The integration style characterized patients curious about their crisis, recognizing continuity in their mental life before, during and after the crisis, which is considered as a positive experience for the future. The integration style is associated to more mature defense mechanisms. On the other hand, the sealing-over style characterized patients viewing their crisis as an interruption in their mental life, not related to their personal difficulties and as an unworthy experience to examine. The sealing-over style is related to maladaptive mechanisms of defense. A dimensional score ranging from 0 to 100 was calculated [42]. Scores higher than 49 reflect “integration”; while scores equal to or lower than 49 indicate “sealing-over” of the emotional crisis.

#### General functioning

The Global Assessment Functioning (GAF) scale describes the current psychological, social and occupational functional status of a patient in a single measure ranging from 0 to 100 [43] with higher scores reflecting better functioning and low symptomatology.

### Procedure

The overseeing institutional Ethics Committee approved the study. The study was completely described to patients who gave their informed written consent before assessment. Clinical psychologists performed the assessment. All of them received formal training in using the BPRS 4.0 [35]. Inter-rater reliability was considered excellent (intraclass coefficient correlation >0.80). Assessments were administered as soon as possible at admission within the first 2 weeks of CIC treatment. To address the effectiveness of crisis intervention, all the patients who gave formal consent were invited to fulfill the assessment once again at discharge. It is also to be mentioned that the DSQ-40 was not included in the initial study protocol and was the object of an addendum that took time to be examined before being accepted.

### Statistical analysis

The normality of the distribution of the variables was verified with the Kolmogorov-Smirnov test. The results suggested violation of the normality for all variables; we thus analyzed the data using non-parametric statistics. Group differences (e.g. gender, diagnosis) were examined with Friedman analyses of variance, the Wilcoxon test for related samples, and the Mann-Whitney U-test for independent samples. Association between variables was computed with Spearman’s rho correlation. Categorical variables were analyzed with the Pearson Chi-Square. Statistical analyses were performed with SPSS version 22.0. The significance level was set at *p* ≤ 0.05 after Bonferroni correction for multiple comparisons in order to minimize the risk of false results. The effect size (ES) was estimated using r (r = z/n^½^) approximate value [44] and interpreted according to usual guidelines [45], where ES is small when ≤0.20, medium when ≤0.50 and large when ≤0.80. As inferential statistical analyses and ES only give an incomplete picture of the effect of crisis intervention, the Reliable Change Index (RCI) [46] was also calculated. This enabled to verify whether changes were due to crisis intervention instead of measure errors. The RCI for each patient and each single variable was calculated correcting for measure error and practice effect. The RCI was calculated with the following formula RCI = [(X1 – X2)/ SE], where X1 and X2 represent patient scores at intake and discharge respectively, and SE (standard error) = SD1*(1 - r)^1/2^, where SD1 = standard deviation of the variable at intake and r = test – retest reliability computed with non-parametric correlation coefficients (Spearman’s rho or Cronbach’s alpha). To classify outcome as improved or deteriorated we chose a criterion of RCI ± |1.96| with at least a 95% confidence interval; when this criterion was not met outcome was considered as undetermined. The outcome of each variable was classified according to these three categories. As recommended in the literature [47, 48], we also reported the proportion of missing data and the percentage of dropouts. In order to examine crisis intervention effectiveness with the maximum of data, we handled the problem of missing data using all the observations per variable only if the patient was administered the BPRS 4.0 at intake and at discharge. The main reason legitimating this choice was the fact that we were studying the psychometric characteristics of the BPRS 4.0 in a sample of patients presenting an emotional crisis [35]. We were also interested in testing whether “Non Attenders” (patients participating only to initial assessment and crisis intervention) differed from “Attenders” (patients participating to both assessments and crisis intervention) because significant differences between these two groups may underpower statistical analyses, constitute a bias in sample selection, and prevent generalization of crisis intervention effectiveness [49].

## Results

### Characteristics of the sample at intake

Baseline assessments (T0) were computed within 12.12 days on average (SD = 11.22) after intake the initial appointment with the psychiatrist and the nurse.

#### Socio-demographic variables

As presented in Table 1, this study included 352 middle-aged patients (mean = 38.56, SD = 10.91), two thirds being women (62.22%), the latter being younger than the men. No other gender differences were found. Only few patients (*n* = 7, 1.99%) referred to the CIC lived in sheltered housings or benefited from accommodations in hotels. Almost two thirds were employed or students while the unemployed benefited from the financial support of social insurances (e.g. social security, unemployment pension or disability pension).

**Table 1:** Socio-demographic characteristics of the sample (*N* = 352)

	Sample (*N* = 352)		Women (*N* = 219)		Men (*N* = 133)		Statistics	*p*
	Mean	SD	Mean	SD	Mean	SD		
Age	38.56	10.91	37.41	11.29	40.45	10.00	Z = −2.69	0.007
	N	(%)	N	(%)	N	(%)		
Living conditions^a^								
Own flat, family, and shared accommodation	341	(98.00)	214	(61.50)	127	(36.50)	χ^2^ = 1.16 (df = 1)	0.28
Sheltered housing	7	(2.01)	3	(0.86)	4	(1.14)		
Occupational status^b^								
Employed/student	223	(66.35)	139	(41.49)	84	(25.07)	χ^2^ = 0.08 (df = 1)	0.77
Unemployed	112	(36.65)	68	(20.29)	44	(13.13)		

Missing data: ^a^ *N* = 4 and ^b^ *N* = 17

#### Crisis context

As shown in Table 2, patients were mainly referred by the emergency unit. The most common symptoms motivating admission were suicidal ideation or suicide attempt, depressed mood, and anxiety. The predominant trigger events of crisis were couple difficulties, work and family relations. It is to be noted that the trigger event was difficult to categorize or unclear for 20% of patients. Women presented more family problems, couple difficulties, and unclear triggers, while men showed more triggers falling under the category labeled “others” (e.g. domestic violence, alcohol abuse…). No other gender difference was found.

**Table 2:** Crisis context at admission for the sample, by gender, and results of gender comparison

	Sample		Women		Men		Statistics	*p*
	N	(%)	N	(%)	N	(%)		
Referred by							χ^2^ = 7.72 (df = 5)	0.10
Emergency unit	164	(46.59)	109	(30.97)	55	(15.63)		
Outpatient unit	33	(9.38)	21	(5.97)	12	(3.41)		
Private practitioner	106	(30.11)	67	(19.03)	39	(11.08)		
Psychiatric hospital	39	(11.08)	17	(4.83)	22	(6.25)		
Self-referred	10	(2.84)	5	(1.42)	5	(1.42)		
Specialized psychiatric units^a^	0	(0.00)	0	(0.00)	0	(0.00)		
Primary problem presented							χ^2^ = 8.11 (df = 7)	0.32
Anxiety	50	(14.20)	28	(7.95)	22	(6.25)		
Delusions/hallucinations	13	(3.93)	6	(17.04)	7	(1.99)		
Depressed mood	112	(31.82)	71	(20.17)	41	(11.64)		
Substance abuse	7	(1.99)	5	(1.42)	2	(0.57)		
Suicidal ideation or attempt	143	(40.63)	92	(26.13)	51	(14.49)		
Violence	5	(1.42)	1	(0.28)	4	(1.14)		
Not reported	17	(4.83)	13	(3.69)	4	(1.14)		
Other	5	(1.42)	2	(0.57)	3	(0.85)		
Trigger event							χ^2^ = 25.75 (df = 5)	< 0.001
Couple	148	(42.05)	101	(28.69)	47	(13.35)		
Family	59	(16.76)	44	(12.50)	15	(4.26)		
Work	69	(19.60)	38	(10.79)	31	(8.81)		
Other	53	(15.06)	20	(5.68)	33	(9.38)		
Unclear	23	(6.53)	16	(4.54)	7	(1.99)		

^a^Given N values this Group was excluded of χ^2^ analyses

#### Main diagnosis and medication

Table 3 summarizes patients’ diagnosis and medication status. The most common main diagnosis was mood disorders (*n* = 253, 72.16%). The next important category of diagnoses was neurotic, stress-related and somatoform disorders (*n* = 76, 21.59%). Other diagnoses represented altogether 6.53% (*n* = 23). No gender differences were found.

**Table 3:** Main diagnosis and medication of the sample (*N* = 352)

	Sample		Women		Men		Statistics	*p*
	N	(%)	N	(%)	N	(%)		
ICD-10 Diagnosis^a^								
F30-39: Mood (affective) disorders	253	(72.16)	155	(44.03)	98	(27.84)	χ^2^ = 5.12 (df = 4)	0.28
F31: Bipolar affective disorder	18	(5.11)						
F32: Depressive episode	131	(37.22)						
F33: Recurrent depressive disorder	104	(29.54)						
F40-F48 Neurotic, stress-related and somatoform disorders	76	(21.59)	50	(14.20)	26	(7.38)		
F40: Phobic anxiety disorders	4	(1.13)						
F41: Other anxiety disorders	9	(2.56)						
F42: Obsessive compulsive disorder	3	(8.52)						
F43: Reaction to severe stress	60	(17.05)						
Others	23	(6.53)	14	(3.98)	9	(2.56)		
F10 Mental and behavioral disorders due to psychoactive substance use	3	(0.85)						
F20 Schizophrenia, schizotypal and delusional disorders;	5	(1.42)						
F50 Behavioral syndromes associated with physiological disturbances and physical factors	3	(0.56)						
F60 Specific personality disorders	11	(3.13)						
Z73 Problems related to life - management difficulties	1	(0.03)						
Medication								
None	32	(9.10)	20	(5.68)	12	(3.40)	χ^2^ = 1.35 (df = 2)	0.51
Monotherapy	61	(17.33)	34	(9.65)	27	(7.67)		
Polytherapy	259	(73.58)	165	(46.87)	94	(26.70)		

^a^As the frequency for number of cells was very low for women and men (not reported), statistics were compute considering the following three categories: F30-39: Mood (Affective) Disorders, F40-F48 Neurotic, stress-related somatoform disorders and Others

Most of the patients (*n* = 320, 90.90%) received psychotropic medication with the majority taking more than one drug. As principal medication, patients were administered antidepressants (*n* = 213, 60.51%), antipsychotics (*n* = 44, 12.50%), hypnotics (*n* = 15, 4.26%), mood stabilizers (*n* = 14, 3.97%), and benzodiazepines (*n* = 11, 3.13%). The other psychotropic medications were administered for less than 1% of the sample. No gender differences were found.

#### Clinical characteristics

Table 4 contains details about the clinical psychiatric features of the sample. Most patients presented severe to extremely severe psychiatric symptoms on both the BPRS 4.0-C and the SCL-90-R-C. These two measures were positively and significantly correlated (*r* = .39, *p* < 0.001). The patients used their defense mechanisms differentially (Friedman χ^2^ = 37.45, df = 2, *p* < 0.001). Post-hoc analyses show that mature defenses were more present than neurotic (Wilcoxon Z = −3.68, *p* < 0.001) and immature defenses (Wilcoxon Z = −6.68, *p* < 0.001), and neurotic defenses were more present than immature defenses (Wilcoxon Z = −4.05, *p* < 0.001). One fifth of the patients showed a sealing-over recovery style to overcome their emotional crisis. The GAF score indicates that patients presented, on average, moderate symptoms of mental illness or moderate functional impairments. None of the comparisons between genders reached statistical significance.

**Table 4:** Psychiatric symptoms, defense mechanisms, recovery styles and social functioning

Variables		Sample		Women		Men		Statistics	*p*
		N	(%)	N	(%)	N	(%)		
Psychiatric symptoms									
BPRS-4.0	No illness^a^	0	(0.00)	0	(0.00)	0	(0.00)	χ^2^ = 0.65 (df = 3)	0.89
	Mild	6	(1.77)	4	(1.17)	2	(0.58)		
	Moderate	27	(7.95)	15	(4.41)	12	(3.52)		
	Severe	71	(20.88)	44	(12.94)	27	(7.94)		
	Extremely severe	236	(69.47)	149	(43.82)	87	(25.58)		
SCL-90-R	No distress	10	(3.65)	4	(1.45)	6	(2.18)	χ^2^ = 5.92 (df = 4)	0.21
	Mild	8	(2.92)	6	(2.18)	2	(0.73)		
	Moderate	16	(5.84)	8	(2.92)	8	(2.92)		
	Severe	32	(11.67)	24	(8.76)	8	(2.92)		
	Extremely severe	208	(75.91)	132	(48.17)	76	(27.73)		
Recovery **(**RSQ)	Sealing over	54	(19.01)	39	(13.73)	15	(5.28)	χ^2^ = 2.78 (df = 1)	0.10
	Integration	230	(80.98)	138	(48.59)	92	(32.39)		
		Mean	SD	Mean	SD	Mean	SD		
Defenses (DSQ-40)	Mature	5.24	1.31	5.21	1.32	5.28	1.32	Z = −0.25	0.80
	Neurotic	4.66	1.41	4.67	1.45	4.65	1.32	Z = −0.11	0.92
	Immature	4.24	1.07	4.18	1.04	4.32	1.11	Z = −0.54	0.59
Global functioning (GAF)		52.51	10.99	52.56	11.21	52.42	10.64	Z = −0.58	0.57

*BPRS-4.0* Brief Psychiatric Rating Scale version 4.0, *SCL-90-R* Symptom Checklist – Revised, *DSQ-40* Defense Style Questionnaire 40 items, *RSQ* Recovery Style Questionnaire, *GAF* Global Assessment Functioning

^a^Given N values this Group was excluded of χ^2^ analyses

### Crisis intervention effectiveness

#### Dropouts

Among the 352 patients, 58.5% skipped the assessment at discharge. Attenders were older (mean = 40.65 years, SD = 11.47) than Non Attenders (mean = 37.08 years; SD = 10.26) (Mann-Whitney Z = −2.81, *p* = 0.005). The proportion of men and women was equivalent in both groups (χ2 = 0.73, df = 1, *p* = 0.39). Regarding “activity and incomes”, Attenders depended less on the social support system (χ2 = 14.7, df = 5, *p* = 0.01). They also presented more obsessive features in SCL-90-R dimensions (Attenders: mean = 1.82, SD = 0.83; Non Attenders: mean = 1.61, SD = 0.82, Mann-Whitney Z = −2.28, *p* = 0.02). No other difference reached statistical significance. Also there was no difference in medication use (χ2 = 1.35, df = 2, *p* = 0.58). Psychopathology and social functioning were similar in both groups (all *p* > 0.10; for details see Additional file 1).

#### Missing data at discharge

Table 5 also provides the number of observations per variable. The highest proportion of missing data at discharge was observed for the DSQ-40 (48.18%), followed by the GAF (22.62%), the RSQ (21.16%) and the SCL-90-R (10.22%).

**Table 5:** Results of the comparisons between intake and discharge

	Intake		Discharge		Statistics Wilcoxon Z value	N	*p*	Effect size
	Mean	(SD)	Mean	(SD)				
Psychiatric Symptoms								
BPRS-4.0								
Reality distortion	1.80	(0.80)	1.53	(0.73)	−3.86	137	0.001*	0.33
Activation	1.42	(0.68)	1.33	(0.56)	−1.56	137	0.119	0.13
Apathy	1.52	(0.55)	1.36	(0.45)	−3.55	137	0.004	0.30
Mood disturbance	3.41	(0.78)	2.45	(0.83)	−8.73	137	< 0.001*	0.74
Disorganization	1.16	(0.34)	1.08	(0.23)	−2.66	137	0.007	0.22
Somatization	2.06	(0.76)	1.63	(0.60)	−5.74	137	< 0.001*	0.49
SCL-90-R								
Somatization	1.40	(0.89)	0.92	(0.94)	−6.10	123	< 0.001*	0.55
Obsession compulsion	1.82	(0.84)	1.21	(0.96)	−6.83	123	< 0.001*	0.62
Interpersonal vulnerability	1.38	(0.92)	0.99	(0.94)	−5.87	123	< 0.001*	0.53
Depression	2.13	(0.83)	1.30	(0.95)	−7.84	123	< 0.001*	0.71
Anxiety	1.52	(0.83)	0.93	(0.86)	−7.18	123	< 0.001*	0.65
Hostility	0.98	(0.87)	0.58	(0.69)	−5.70	123	< 0.001*	0.52
Phobic anxiety	0.97	(0.83)	0.68	(0.85)	−4.42	123	< 0.001*	0.40
Paranoid	1.38	(0.95)	1.00	(0.93)	−5.67	123	< 0.001*	0.52
Psychoticism	0.82	(0.61)	0.58	(0.66)	−5.32	123	< 0.001*	0.49
Defense mechanisms								
DSQ-40								
Mature	5.31	(1.30)	5.81	(1.49)	−2.98	71	0.004	0.36
Neurotic	4.55	(1.41)	4.37	(1.14)	−1.14	71	0.260	0.14
Immature	4.17	(1.03)	3.83	(0.93)	−3.24	71	0.001*	0.39
Recovery Style								
RSQ	63.31	(17.15)	70.70	(15.44)	−4.79	108	< 0.001*	0.47
Global functioning								
GAF	52.46	(11.07)	63.00	(12.74)	−6.78	106	< 0.001*	0.66

*BPRS-4.0* Brief Psychiatric Rating Scale version 4.0, *SCL-90-R* Symptom Checklist – Revised, *DSQ-40* Defense Style Questionnaire 40 items, *RSQ* Recovery Style Questionnaire, *GAF* Global Assessment Functioning

*p < 0.05 after Bonferroni correction for multiple comparison

#### Length of intervention

The crisis intervention lasted on average 57 days (median) [minimum =11; maximum =409 days].

#### Intake vs discharge results comparisons

Table 5 summarizes the statistical results of the comparisons. After crisis intervention there was a significant reduction of symptom severity on the BPRS 4.0 and SCL-90-R dimensions, except for the Activation dimension of the BPRS 4.0. There was also a significant decrease of immature defense mechanisms and a greater use of mature mechanisms, whereas neurotic defenses remained stable (DSQ-40). Patients adopted a more integrative recovery style (RSQ) and had a better global functioning (GAF). All the comparisons survived the conservative Bonferroni correction for multiple comparisons, except the apathy and disorganization BPRS 4.0 factors and the mature defense mechanisms (DSQ-40). None of the improvements were related to gender (all *p* > 0.10).

#### Effect sizes

As reported in Table 5, the crisis intervention had medium effect sizes for all variables except for the Activation and Disorganization dimensions of the BPRS 4.0 where the magnitude of the effect size was small. Also to be mentioned, the highest magnitude was found in observer mood disturbance and depression variables.

#### Reliability Change Index (RCI)

Table 6 summarizes the RCI for each variable. After crisis intervention, the clinical evolution fell into the undetermined category for most patients, with proportions ranging from 41.46% to 96.35%; some patients clinically deteriorated, with proportions ranging from 0.81% to 18.31% and some improved with proportions ranging from 2.19% to 52.03%.

**Table 6:** Clinical evolution using the reliability change index

	Deteriorated		Undetermined		Improved	
	N	(%)	N	(%)	N	(%)
Psychiatric symptoms						
BPRS-4.0						
Reality distortion	5	(3.65)	132	(96.35)	0	(0.00)
Activation	5	(3.65)	122	(89.05)	10	(7.30)
Apathy	3	(2.19)	124	(90.51)	10	(7.30)
Mood disturbance	5	(3.65)	62	(45.26)	70	(51.09)
Disorganization	11	(8.03)	123	(89.78)	3	(2.19)
Somatization	3	(2.19)	110	(80.29)	24	(17.52)
SCL-90-R						
Somatization	15	(12.20)	70	(56.91)	38	(30.89)
Obsession compulsion	1	(0.81)	79	(64.23)	43	(34.96)
Interpersonal vulnerability	5	(4.07)	79	(64.23)	39	(31.71)
Depression	8	(6.50)	51	(41.46)	64	(52.03)
Anxiety	3	(2.59)	64	(55.17)	49	(42.24)
Hostility	2	(1.77)	77	(68.14)	34	(30.09)
Phobic anxiety	7	(5.69)	95	(77.24)	21	(17.07)
Paranoid	1	(0.81)	99	(80.49)	23	(18.07)
Psychoticism	3	(2.65)	61	(53.98)	49	(43.36)
Defense mechanisms						
DSQ-40						
Mature	5	(7.04)	44	(64.79)	20	(28.17)
Neurotic	13	(18.31)	46	(67.61)	10	(14.08)
Immature	13	(18.31)	50	(73.24)	6	(8.45)
Recovery Style						
RSQ	7	(6.48)	55	(58.33)	38	(35.19)
Global functioning						
GAF	4	(3.70)	65	(60.19)	39	(36.11)

*BPRS-4.0* Brief Psychiatric Rating Scale version 4.0, *SCL-90-R* Symptom Checklist – Revised, *DSQ-40* Defense Style Questionnaire 40 items, *RSQ* Recovery Style Questionnaire, *GAF* Global Assessment Functioning

## Discussion

In this paper we presented the findings of a naturalistic study that took place in a Crisis Intervention Center (CIC). In particular, we aimed to characterize the socio-demographical and clinical profiles of the patients referred to the CIC in a context of emotional crisis and participating in intensive treatment, as well as assessing the effectiveness of the latter.

### Sample characteristics at intake

Patients were middle aged, with a higher proportion of women. The majority of patients was addressed to the CIC by the emergency unit of the hospital or by private practitioners which is in accordance with previous findings [15, 22, 50, 51, 53], because the patients had attempted to commit suicide, had suicidal ideation or presented depressed mood due to relational difficulties either in their family, in their couple or at their work place. Most of the patients were diagnosed as presenting a depressive or neurotic disorder with low global functioning and were administered a polypharmacological treatment. Regarding socio-demographic variables and crisis features, our findings are consistent with those reported in previous studies conducted in very similar psychiatric services providing crisis treatment [9, 14, 15, 17, 50–53]. Nevertheless, we found a higher percentage of working patients and student patients than in other studies [22] as well as patients on average 10 years older than those from Marini’s study [50]. Moreover, unlike previous studies [9, 23, 52], our sample almost did not include patients with schizophrenia and related psychotic disorders. As previously observed, the psychiatric symptoms are severe [9, 15, 23, 50]. As none of the previous studies assessed the defense mechanisms in patients with emotional crisis, we cannot compare our results and we can only state that at the beginning of the treatment patients used preferentially mature defense mechanisms, followed by neurotic and immature defense mechanisms. Regarding the recovery styles, the integration style was common in patients with affective disorders, which is in accordance with previous observations [54]. These last two observations seem contrary to the psychodynamic theory of crisis [27] and suggest that emotional crisis does not have the deleterious impact expected on psychological functioning.

### Dropouts and missing data

The attrition rate and missing data were high. The proportion of dropouts found herein fell in the upper range, that is 15% to 60%, of premature termination reported in the literature [55]. However, previous studies conducted in similar crisis centers reported lower rates of premature termination, i.e. from 16% to 37.5% [14, 50, 51]. This difference may be explained by the fact that patients having already started to work before the end of the crisis intervention may have found irrelevant to come back to the CIC for research purposes. Nevertheless, no significant difference between attender and non-attender groups was found and, missing data having mostly occurred at random, we can assume that drop-outs and missing data have only slightly biased effects on the findings [49].

### Crisis intervention efficiency

The crisis intervention lasted on average 8 weeks, but there was a considerable variation among patients, ranging from 2 to 58 weeks. This variation likely occurred because of the complexity of the cases associating psychiatric symptom severity and social problems (e.g. homelessness or dismissal occurring during intervention…).

Findings of this study suggest effectiveness on different levels of a time-limited intensive psychodynamic oriented crisis intervention. First, in terms of symptom reduction, a change of the defense mechanisms towards more mature ones was found, possibly as a better integration of the crisis as well as an improvement of the general functioning occurred. However, the effect size and reliable change index tone down a bit the high statistical differences observed after the crisis intervention. Symptom reduction and improvement in social functioning are in accordance with the findings of previous studies underwent in care units including aspects of crisis intervention [15, 24, 25, 52]. Nevertheless, to the best of our knowledge, this is the first study demonstrating a significant change of the predominant defenses mechanisms and change in recovery style; therefore, comparisons with other studies were not possible.

### Scope of the findings for clinical practice

The results reported here might have important clinical implications. The socio-demographic and clinical profile of the patients at intake in a Crisis center may help the clinician to offer the patients tailored pharmacological and psychological (e.g. individual or group interventions) treatments. They also provide evidence that crisis intervention should not only address psychiatric symptoms - especially suicidal thoughts and behavior - and psychopathology in general, but also promote psychological adjustment to the emotional crisis (e.g. increasing mature defense mechanisms and integration recovery style) as well as social functioning and prevent future decompensations. They also support the view that family and couple psychotherapy should also be considered as crucial ingredients in crisis intervention. In addition, it is important to note that patients may benefit from a specific treatment approach according to their recovery style [41, 56–59].

### Limitations

The generalization of our findings regarding crisis intervention effectiveness is restricted due to several limitations that need to be acknowledged. The naturalistic design of the study did not allow to control whether improvements were due to the natural course of emotional crisis resolution. The high percentage of dropouts suggested that psychodynamically oriented crisis intervention is not suitable for all patients. Moreover, we could not differentiate crisis intervention dropouts (patients terminating prematurely the intervention) from study dropouts (patients participating in the intervention but withdrawing from the study). The length of intervention was neither defined nor controlled for. Only patients entering the intensive crisis program and willing to participate in the study were included. Thus, it is possible that the profile of these patients may be different to the one of the patients addressed to the CIC. It is also to be mentioned that psychiatric diagnosis was not established using a structured interview such as the SCID [60] or MINI [61, 62]. Moreover, the clinical assessments used did not take into account aversive past life events, which could be related to several psychiatric disorders and considered as a pivotal ingredient in the current emotional crisis [63–65]. Comorbidities (e.g., severe medical conditions, substance abuse) were not considered. Moreover, follow-up assessment at several time points (e.g. 6 and 12 months), which is common in psychotherapy research [66, 67], was not used. Another limitation is the absence of differentiation between first and multiple episode crises which probably need different interventions [17]**.** As there are no normative data for the general population regarding several instruments (e.g. BPRS 4.0, DSQ-40, RSQ, and GAF), Clinical Significance could not be applied [46], and thus the clinical outcome was not fully examined. Moreover, the impact of staff members’ framework and adherence to rigorous crisis concepts was not considered. Finally, adverse effects occurring during intervention (e.g. suicide attempts, suicide, scarification, hospitalization, violent behaviours...) were not recorded.

## Conclusion

In summary, patients facing an emotional crisis do not form a homogenous group, regarding e.g. crisis context, diagnosis, symptom severity. As a consequence, specific brief family therapy, couple therapy or suicide intervention should be viewed as adjunctive treatments for most of the individuals referred to a crisis intervention center. This also has implications for crisis intervention staff members who may benefit from continuous supervision and specific training (e.g. suicide intervention skills training). This study suggests that brief psychodynamically-oriented crisis interventions in specific units like the CIC may contribute to alleviate symptoms, to change defense mechanisms, as patients present more mature defenses mechanisms after treatment, to increase global functioning, and to enhance the patients’ psychological adjustment to their emotional crisis towards a more “integrative” recovery style.

Findings reported herein provide a basis for our future work on the generalization of the study to other crisis centers and on the effectiveness of brief intensive crisis treatment. Further research is needed to clarify whether the improvements are clinically significant and remain stable on the long run.

## Additional file

Additional file 1: Table: Comparisons between Admission Assessment Non-Attender and Attender Groups. (DOCX 18 kb)

## Abbreviations

BPRS 4.0: Brief psychiatric rating scale version 4.0
BPRS 4.0-C: Brief psychiatric scale version 4.0 – categories
CIC: Crisis intervention center
DSQ 40: Defense style questionnaire
EMDR: Eye movement desensitization and reprocessing
ES: Effect size
GAF: Global assessment functioning
ICD-10: International Classification of Diseases, 10th revision
M: Mean
MINI: Mini International Neuropsychiatric Interview
RCI: Reliable change index
RSQ: Recovery style questionnaire
SARS: Severe acute respiratory syndrome
SCID: Structural clinical interview for DSM
SCL-90-R: Symptom checklist 90 revised
SCL-90-R-C: Symptom checklist 90 revised – categories
SD: Standard deviation
SE: Standard error
SPSS: Statistical package for social sciences
X: Patients scores
