# Supplementary material for: Emotional crisis in a naturalistic context: characterizing outpatient profiles and treatment effectiveness
Source: BMC Psychiatry. 2017 Apr 7;17:130. doi: 10.1186/s12888-017-1293-3 (PMC5384152; doi:10.1186/s12888-017-1293-3)
Supplement: Additional file 1: — Table: Comparisons between Admission Assessment Non-Attender and Attender Groups. (DOCX 18 kb) [file 12888_2017_1293_MOESM1_ESM.docx]

Additional file 1

Table: Comparisons between Admission Assessment Non-Attender and Attender Groups

Non-Attenders Attenders Statistics

Mean (SD) Mean (SD) Wilcoxon N *p*

Z value

Psychiatric Symptoms

BPRS-4.0

Reality distortion 1.79 (0.86) 1.80 (0.80) -0.34 341 0.73

Activation 1.41 (0.66) 1.42 (0.69) -0.43 341 0.67

Apathy 1.65 (0.74) 1.51 (0.55) -1.24 341 0.22

Mood disturbance 3.55 (0.80) 3.39 (0.79) -1.80 341 0.07

Disorganization 1.22 (0.39) 1.16 (0.35) -1.93 341 0.05

Somatization 1.96 (0.67) 2.06 (0.77) -1.18 341 0.24

SCL-90 R

Somatization 1.41 (0.92) 1.40 (0.90) -0.13 275 0.90

Obsession compulsion 1.62 (0.82) 1.80 (0.83) -1.90 275 0.06

Interpersonal vulnerability 1.36 (0.80) 1.40 (0.93) -0.02 275 0.98

Depression 1.97 (0.84) 2.13 (0.83) -1.58 275 0.11

Anxiety 1.49 (0.78) 1.52 (0.83) -0.40 275 0.69

Hostility 1.04 (0.90) 1.00 (0.87) -0.24 275 0.81

Phobic anxiety 0.98 (0.88) 0.95 (0.81) -0.11 275 0.91

Paranoid 1.28 (0.88) 1.38 (0.94) -0.69 275 0.49

Psychoticism 0.79 (0.62) 0.83 (0.62) -0.55 275 0.58

Global severity index 1.41 (0.63) 1.46 (0.64) -0.80 275 0.43

Defense mechanisms

DSQ-40

Mature 5.26 (1.35) 5.23 (1.27) -0.35 165 0.73

Neurotic 4.71 (1.41) 4.60 (1.40) -0.60 165 0.55

Immature 4.25 (1.08) 4.21 (1.05) -0.25 165 0.81

Recovery Style

RSQ 64.21 (14.23) 63.14 (15.91) -0.48 285 0.63

Global functioning

GAF 52.44 (11.03) 52.67 (10.96) -0.50 264 0.62

BPRS-4.0 = Brief Psychiatric Rating Scale version 4.0; SCL-90-R = Symptom Checklist – Revised; DSQ-40 = Defense Style Questionnaire 40 items; RSQ = Recovery Style Questionnaire; GAF = Global Assessment Functioning
